# Fabrication of Cu_2_Sn_1-x_Ge_x_S_3_ Thin-Film Solar Cells via Sulfurization of Cu_2_GeS_3_/Cu_2_SnS_3_ Stacked Precursors

**DOI:** 10.3390/ma17081886

**Published:** 2024-04-19

**Authors:** Takeshi Tasaki, Kazuo Jimbo, Daiki Motai, Masaya Takahashi, Hideaki Araki

**Affiliations:** National Institute of Technology (KOSEN), Nagaoka College, Nagaoka 940-8532, Japan

**Keywords:** Cu_2_Sn_1-x_Ge_x_S_3_, thin films, solar cells, co-evaporation method

## Abstract

Cu_2_Sn_1-x_Ge_x_S_3_ (CTGS) is a compound composed of relatively abundant elements in the crust of the earth. The band gap of CTGS can be tuned by substituting elements at the Sn and Ge sites, making it an attractive material for low-environmental-impact solar cells. In this study, CTGS thin films were fabricated with a controlled [Ge]/([Ge] + [Sn]) composition ratio (x) by combining the co-evaporation method and sulfurization in an infrared furnace. Furthermore, the effect of Na on the CTGS and changes in the solar cell properties were investigated by stacking and sulfurizing NaF on the precursor fabricated using the co-evaporation method. As a result, CTGS with varying x was successfully fabricated by varying the deposition time of the Cu_2_GeS_3_ layer using co-evaporation. Additionally, CTGS prepared by doping with Na showed enlarged CTGS crystals compared to Na-free CTGS. The fabricated CTGS solar cells achieved a power conversion efficiency of more than 4.5% after doping with Na.

## 1. Introduction

Cu_2_Sn_1-x_Ge_x_S_3_ (CTGS) is a solid solution of Cu_2_SnS_3_ (CTS) and Cu_2_GeS_3_ (CGS) and is a promising candidate material for next-generation solar cells. CTS is a p-type semiconductor with a band gap energy of approximately 0.9 eV and a high optical absorption coefficient of more than 10^4^ cm^–1^ in the visible to the near-infrared region [1]. CGS has been reportedly fabricated in bulk crystals [2], and its band gap energy is estimated to be approximately 1.5 eV [3]. By varying the Ge composition ratio (x), the band gap of CTGS can be tuned almost linearly between 0.9 and 1.5 eV in bulk crystals [4,5,6] and nanoparticles [7], making it suitable as a light-absorbing layer in solar cells. Furthermore, the constituent elements are inexpensive and non-toxic compared to the leading compounds in solar cells, such as Cu(In,Ga)(S,Se)_2_, CdTe, and Cu_2_ZnSn(S,Se)_4_. In addition, excellent atmospheric stability has been reported in CTS-based solar cells [8]. Therefore, solar cells composed of CTGS, which is similar to CTS, are expected to have advantages over perovskite solar cells [9,10], which have been the focus of recent attention, in that they do not contain Pb and are more stable in air. Consequently, various compositions and fabrication processes have been reported for CTGS thin-film solar cells, including CTS cells corresponding to x = 0 and CGS thin-film solar cells corresponding to x = 1. As shown in Table 1 [11,12,13,14,15,16,17,18,19,20], a type of CTGS with x = 0 (i.e., CTS), achieved a power conversion efficiency (PCE) of more than 5% [11,12]. PCEs exceeding 5% have also been reported in CTGS solar cells fabricated by alloying Ge with CTS in thin films fabricated by the sulfurization of Ge/Cu–SnS_2_ precursors with S and SnS_2_ [14] and in thin films obtained by the sulfurization of Cu–Sn metal precursors with S and GeS_2_ vapor [15,16]. In particular, the highest reported PCE of 6.7% was obtained for CTGS-based solar cells using a gradient band gap structure formed by varying x of CTGS in the depth direction of the thin film [16]. Similarly, we previously reported several CTGS thin films fabricated using the co-deposition method, including CTS thin-film solar cells with x = 0 [21,22,23,24,25]. However, none of these CTGS efficiencies are sufficient for practical use, as the PCE does not reach above 20% as in practical Cu(In,Ga)(S,Se)_2_ and CdTe solar cells [26,27]. In this work, to improve the efficiency of CTGS solar cells, a co-deposition method using a Knudsen cell was employed, which allows precise control of the deposition source temperature. This method can control a wide range of complex processes and compositions, such as the three-stage process (deposition rate vs. time control) used for CIGS film formation [28]. Thus, the advantages include the ease of switching deposition conditions, such as depositing a CTS layer immediately after the CGS layer, and the study of appropriate stacking conditions. Using this co-evaporation method, we prepared a stacking precursor of the Cu_2_GeS_3_/Cu_2_SnS_3_ structure and fabricated dense, flat, high-quality CTGS thin films by sulfurizing the obtained precursor films. We attempted to fabricate CTGS thin films using CGS/CTS stacking precursors as a method different from that employed in the previous study, in which single-layer precursor thin films were prepared by the co-deposition of CTGS thin films. Furthermore, CTS thin-film solar cells with Na doping by the sulfurization of NaF-deposited precursor thin films have been reported to exhibit improved solar cell characteristics [11,12]. Therefore, we also investigated the effect of Na on CTGS by combining precursor fabrication using the co-evaporation method and sulfurization with NaF stacking and evaluated the resulting changes in the CTGS solar cell’s properties.

## 2. Materials and Methods

The following materials were procured: Mo disc (99.95%, 3 mm thick, Fruuchi Chemical Corporation, Tokyo, Japan), Cu shot (99.9999%, Fruuchi Chemical Corporation, Japan), Sn shot (99.9999%, Fruuchi Chemical Corporation, Japan), Ge shot (99.999%, Fruuchi Chemical Corporation, Japan), sodium fluoride (NaF, 99%, Nacalai tesque, Inc., Kyoto, Japan), S chunk (99.9999%, Fruuchi Chemical Corporation, Japan), cadmium iodide (CdI_2_, 99%, FUJIFILM Wako Pure Chemical Corporation, Osaka, Japan), Thiourea (CS(NH_2_)_2_, 98%, Nacalai tesque, INC., Kyoto, Japan), ammonia water (NH_3_, 28 wt%, Fujifilm Wako Pure Chemical Corporation, Osaka, Japan), ZnO: Al sputter target (Al_2_O_3_, 2 wt%, 3 mm thick, 99.99%, Fruuchi Chemical Corporation, Japan), and Al (99.99%, Niraco Corporation, Tokyo, Japan).

**Figure 1:**
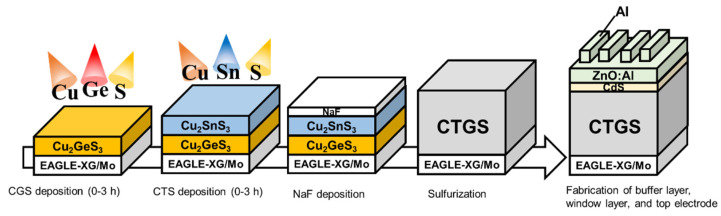
Schematic of the CTGS-based solar cell fabrication process.

Figure 1 shows a schematic of the CTGS solar cell fabrication process. The Mo back-contact film is formed on an alkali-free glass substrate EAGLE-XG using DC sputtering. The sputtering conditions are as follows: deposition time, 25 min; applied current, 1.0 A; process pressure, 0.4 Pa; and Ar flow, 20 sccm. CGS and CTS thin films were deposited using a Cu, Ge, Sn, and S co-deposition system (EIKO Co., Tokyo, Japan). The S flux was activated by thermal cracking at 800 °C with S evaporated at 150 °C using the needle valve cracker cell as the S source. The Cu, Sn, and Ge K-cells and the valved cell for S were arranged such that the distance between the aperture position of the cells and the center of the substrate was 215 mm. The CGS layer was prepared by evaporating Cu and Ge with S flux on a Mo-coated substrate under a growth chamber pressure of 10^−4^ Pa. The evaporation rates for Cu and Ge were 0.22 and 0.35 Å/s, respectively. The deposition time *t*_CGS_ of the CGS layer was 0.0–1.5 h. *t*_CGS_ = 0.0 means that the film is not deposited with CGS. Thus, it is a sample fabricated by depositing only the CTS layer. Subsequently, a CTS layer was prepared by depositing Cu and Sn in S flux on the CGS layer. The Sn evaporation rate was 0.35 Å/s. The evaporation time of the CTS layer was varied based on the preceding CGS evaporation time such that the total time for both layers was 3 h. The composition ratio of the fabricated CGS/CTS stacked precursor was measured using wavelength-dispersive X-ray fluorescence (XRF) analysis (ZSX Primus IV, Rigaku, Tokyo, Japan). The NaF layer was deposited on the precursor via thermal evaporation using 10 mg NaF as an evaporation source. Similarly, for comparison, precursors without NaF deposition were prepared. The precursor was placed in a carbon susceptor containing 100 mg of S and sulfurized in an infrared furnace. The sulfurization procedure was implemented by increasing the temperature to 570 °C at a rate of 2 °C/s for 10 min, which was followed by cooling at room temperature. To remove the water-soluble impurities from the sulfurized CTGS thin film, it was immersed in 50 mL of deionized water for 30 min. The composition of these CTGS films was characterized using XRF. The crystal structure was analyzed by performing X-ray diffraction (XRD; MiniFlex, Rigaku, Japan). The surface and cross-sectional morphologies of these thin films were observed using scanning electron microscopy (SEM) (JSM-6060LV, JEOL, Tokyo, Japan). An n-type CdS layer was deposited on the obtained CTGS thin film via chemical bath deposition, and n-type ZnO: Al was prepared via RF sputtering. The reaction conditions for the chemical bath deposition method were previously reported [23]. Here, the CdS buffer layer was an n-type semiconductor with a thickness of approximately 90 nm and was used to form a p-n junction with proper band alignment with CTGS, which was a p-type semiconductor. The transparent conducting oxide layer, ZnO: Al, transmitted light to the CTGS/CdS layer and conducted the photogenerated electrons to the top Al electrode. Finally, solar cells with an EAGLE-XG/Mo/CTGS/CdS/ZnO:Al/Al structure were fabricated by evaporating Al top contacts, and the current density–voltage characteristics were measured using a Xe lamp type solar simulator (SX-UI 500XQ) under irradiation (AM 1.5G and 100 mW/cm^2^).

## 3. Results and Discussion

Table 2 shows the composition ratio x = [Ge]/([Ge] + [Sn]) (hereafter denoted as x) values evaluated via XRF analysis using the CGS/CTS stacked precursor and the thin films obtained via sulfurization. In all thin films, the [Cu]/([Ge] + [Sn]) composition ratio was Cu-poor below the stoichiometric composition ratio of 2.0.

**Figure 2:**
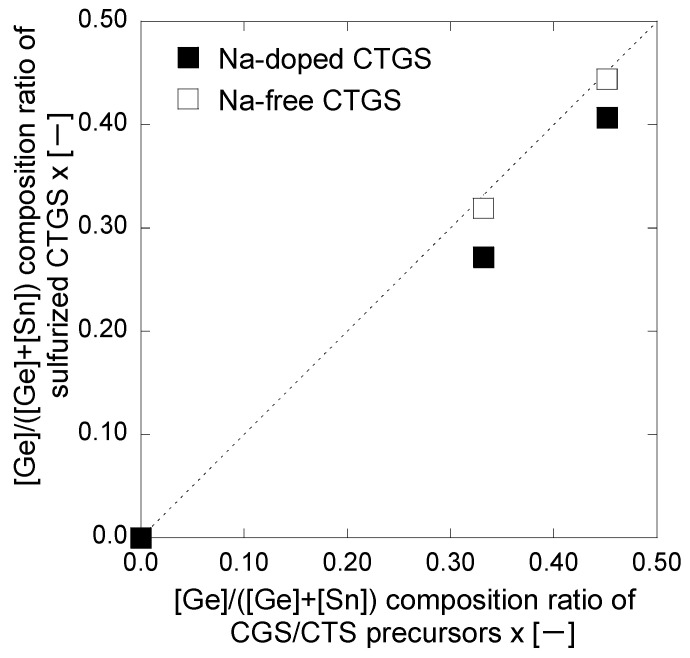
Relationship between x before and after sulfurization of obtained thin films. The horizontal axis represents x before sulfurization, and the vertical axis represents x after sulfurization. The dotted line represents the case in which no difference in composition exists between before and after.

In CTGS, Cu-rich compositions are not suitable for solar cell applications because they induce the formation of short-circuiting CuS [1,29]. The [Cu]/([Ge] + [Sn]) composition ratios obtained in this study are all Cu-poor compositions suitable for solar cell applications. The [Ge]/([Ge] + [Sn]) x ratio increased with increasing *t*_CGS_. However, x in the sulfurized thin film was slightly lower than in the precursor in both cases. Figure 2 shows the behavior of x before and after sulfurization.

If the composition of the precursor thin film before sulfurization and that of the CTGS thin film after sulfurization are the same (i.e., unchanged), then the plot of the marker overlaps the dotted line in Figure 2. However, in the presented results, the marker is plotted below the dotted line because the composition before sulfurization is different from that after sulfurization. This behavior indicates that the [Ge]/([Ge] + [Sn]) composition ratio has decreased due to sulfurization. The Na-doped CTGS showed a remarkable decrease in x after sulfurization compared to the Na-free CTGS. This result suggests that sulfurization may have re-evaporated Ge-containing compounds. GeS with a higher vapor pressure than SnS is a candidate compound that likely causes re-evaporation [30,31]. Conversely, in the present study, S desorption was avoided by annealing with S lumps. As evidenced, the composition ratio [S]/([Cu] + [Ge] + [Sn]) after sulfurization was greater than the stoichiometric ratio of 1.0 in all thin films. The x = 0.00 thin film of Na-free CTGS could not be measured because it was largely peeled off during immersion in deionized water.

**Figure 3:**
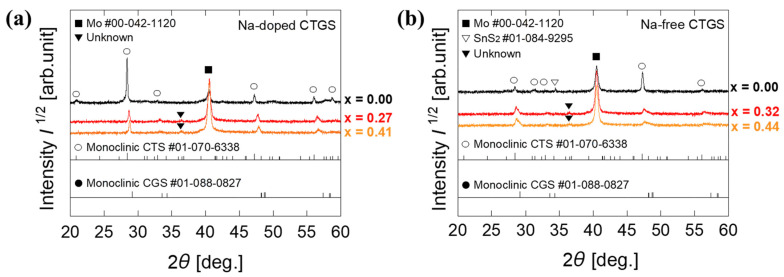
(**a**) XRD spectra for Na-doped CTGS samples with varying values of x. (**b**) XRD spectra for Na non-doped CTGS with varying x.

Figure 3 shows the XRD results of the Na-doped and Na-free CTGS thin films. Figure 3a depicts the XRD spectra of Na-doped CTGS, where a peak near the diffraction peak of CTS with a monoclinic structure (ICDD Powder Diffraction File (PDF) #01-070-6338) is observed. Peaks attributable to monoclinic CTS are observed for the sample with x = 0.00, whereas peaks at x = 0.27 and 0.41 are observed at an angle slightly higher than in CTS and lower than in CGS (PDF#01-088-0827). This result is suggested to be due to a decrease in the lattice parameter caused by the substitution of Ge for a part of Sn in CTS, indicating the formation of CTGS. A small peak is evident around 36.4° for the x = 0.27 and 0.44 samples, but no corresponding peak is observed for the x = 0 sample, suggesting the possibility of an impurity phase. Figure 3b shows the XRD spectra of the Na-free CTGS. The peak at x = 0.00 is attributable to CTS with a monoclinic structure. For the samples at x = 0.32 and 0.44, diffraction peaks are observed at angles slightly higher than that of CTS and lower than that of CGS (PDF#01-088-0827). This result is suggested to be due to a decrease in the lattice constant due to the substitution of Ge for some of the Sn in CTS by the formation of the solid solution CTGS. Furthermore, these peaks for the Na-free CTGS are broader than those in the Na-doped sample, suggesting that the [Ge]/([Ge] + [Sn]) composition ratio x in the thin film is not unity in the Na-free sample compared to the Na-doped films and that the composition distribution in the film is larger. In the Na-free CTGS thin film at x = 0.00, a peak is found around 34° that is not observed in the Na-doped sample. This peak near 34° is attributed to SnS_2_ (PDF #01-084-9295), which can be ascribed to the Cu-poor composition of the precursor. In addition, unattributed peaks were observed at 36° for x = 0.32 and 0.44, which is similar to the Na-doped sample.

**Figure 4:**
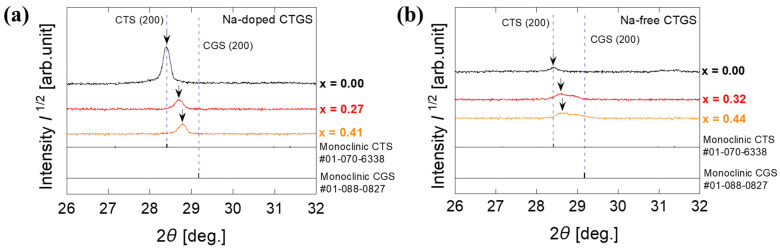
(**a**) Magnified XRD spectra near the (200) plane of monoclinic-CTS and monoclinic-CGS in Na-doped CTGS and (**b**) magnified XRD spectra of Na-free CTGS. Arrows indicate the position of the maximum peak intensity of the diffraction peaks corresponding to the (200) plane in CTS and CTGS.

Figure 4 shows the magnified XRD spectra near the (200) planes of CTS and CGS in the monoclinic structure. Figure 4a depicts the XRD spectra of the Na-doped CTGS in which the peak shifts to a higher angle as x increases. This behavior implies that the lattice of CTGS shrinks as the amount of Ge-substituted Sn sites in CTS increases. Figure 4b presents the magnified XRD spectrum of the Na-free CTGS. The diffraction peak of the thin film with x = 0 coincides with the peak of CTS in the monoclinic structure. However, for x = 0.32 and 0.44 thin films, broad peaks are observed at angles between those expected for CTS and CGS. This finding indicates that the CTGS thin films do not have a single composition and contain regions of different Ge composition ratios. The peaks of the Na-doped sample are sharper than those of the Na-free sample, indicating that sulfurization with Na doping promotes a single composition of Ge in CTGS. The diffraction angle of the maximum diffraction peak intensity is slightly higher at x = 0.44 than at x = 0.32. As with the Na-doped sample, a decrease in the lattice constant with the increasing Ge composition ratio in the sample is suggested.

**Figure 5:**
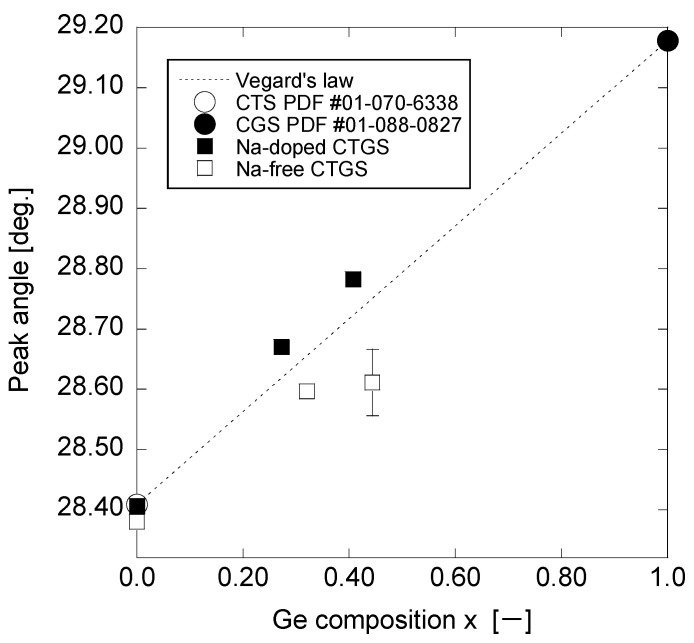
Open and closed circles represent the angles of the peaks corresponding to the (200) plane of the CTS (PDF #01-070-6338) at x = 0 and the CGS (PDF #01-088-0827) at x = 1, respectively. The dotted line connecting them represents the expected diffraction angle of the (200) plane relative to the Ge composition x-value based on Vegard’s law. The open and closed squares represent the diffraction angles of the peak corresponding to the (200) plane of Na-free CTGS and Na-doped CTGS relative to the Ge composition x-value, respectively.

Figure 5 presents the diffraction angles of the maximum-intensity peaks, corresponding to the (200) plane of CTGS, as functions of the Ge composition (x) values. In the case of the Na-free CTGS sample, the diffraction peak of the (200) plane detected at x = 0.44 is broad, and the peak top is not clear; thus, an error bar has been included. The peak angles for Na-doped CTGS and Na-free CTGS show a trend of linear shift with increasing x, which is consistent with Vegard’s law. Therefore, the Ge composition can be controlled by sulfurization of the CGS/CTS stacking precursor with varying *t*_CGS_ with or without Na.

**Figure 6:**
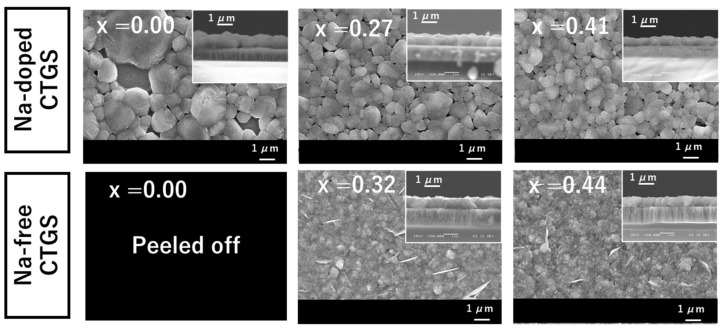
Surface and cross-sectional SEM images of Na-doped and Na-free CTGS.

Figure 6 shows the surface and cross-sectional SEM images of the fabricated CTGS thin films after sulfurization. The surfaces of the Na-free CTGS films exhibit fine crystal grains. Thin plate-like compounds are observed on the surfaces of the CTGS thin films with x-values of 0.32 and 0.44. The observation of similar plate-like morphology has been reported in SnS and SnS_2_ deposition [32,33]. After sulfurization, the concentration of Cu in Na-free CTGS was lower than that in Na-doped CTGS, and SnS_2_ was detected at x = 0.00, as shown Figure 3b. These results and the similar morphologies suggest that the plate-like segregation observed on the surfaces of the samples at x = 0.32 and 0.44 may be assigned to a small amount of SnS and/or SnS_2_ that cannot be detected by XRD. Significant crystal growth is observed in the Na-doped CTGS films compared to that in the Na-free CTGS films. The Na-doped CTGS thin film with x = 0.00 shows higher crystal growth, which is consistent with previous reports of CTS [11,23,34]. Similarly, the growth of CTGS crystals is enhanced in the thin films with x-values of 0.27 and 0.41, suggesting that Na plays a positive role in promoting the growth of CTGS crystals. For CTS thin films, the addition of Na has been reported to enlarge the grains of the thin film significantly, and with the help of NaF, a monoclinic CTS structure has reportedly been formed at a lower substrate temperature than without Na addition [34]. Similarly, the addition of Na to the CTGS thin film promotes the growth of crystal grains in the CTGS film during the heat-treatment process, and the CTS and CGS microcrystals in the CGS/CTS stacked precursor film form CTGS. In addition, the grain boundaries reduce as the crystals grow into large crystal grains with monoclinic structures. In this case, the different phases (SnS_2_, etc.) are consumed as raw materials for the growth of large crystals grain or re-evaporate owing to heat treatment. However, the detailed mechanisms underlying the growth of Na-added CTS and CTGS thin-film crystals remain obscure, and further investigations are required. The voids observed in the Na-doped CTGS thin films may be attributed to the re-evaporation of compounds with high vapor pressures, such as SnS and GeS.

**Figure 7:**
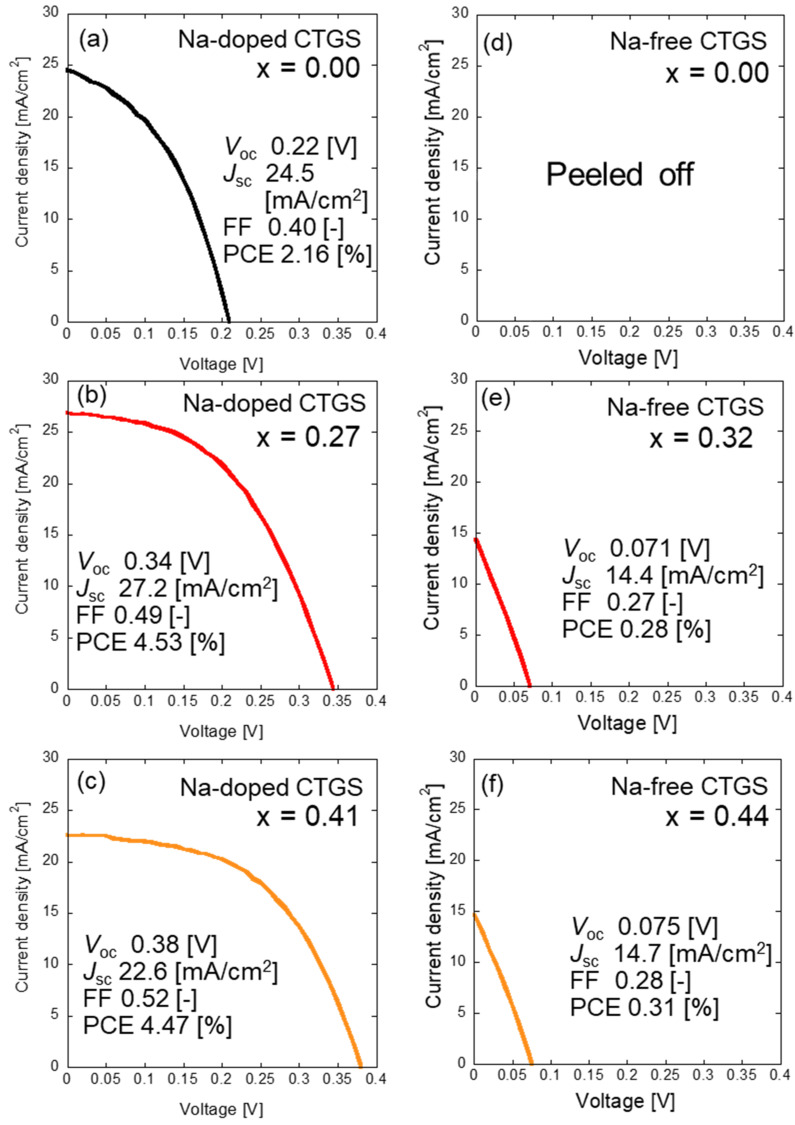
*J*–*V* curves of the cells showing the best photovoltaic characteristics among the CTGS solar cells fabricated for each condition. Photovoltaic properties of (**a**–**c**) Na-doped CTGS solar cells and (**d**–**f**) Na-free CTGS solar cells.

The photovoltaic properties of the solar cells fabricated using these CTGS thin films were studied in detail. Figure 7 shows the measured current density–voltage (*J*–*V*) properties of a solar cell based on the fabricated CTGS. The open-circuit voltage (*V*_oc_) and short-circuit current density *(J*_sc_) of Na-doped CTGS, shown in Figure 7a–c, are significantly higher than those of Na-free CTGS, shown in Figure 7d–f. This trend is similar to the reported effect of Na doping on CTS solar cells [23]. The poor photovoltaic properties of the Na-free CTGS solar cells could be attributed to the presence of plate-like impurities on the surface (observed in the SEM images), which induce carrier recombination at the p-n interface. Additionally, bulk recombination at the CTGS grain boundaries and/or defects in the CTGS grains possibly contribute to the observed poor performance of the device. In contrast, no impurity is visible on the surface of the Na-doped CTGS thin film. The improved electromotive force characteristics of the device may be attributed to the reduction in photo-excited carrier recombination losses due to the reduction in the grain boundaries during the crystal growth.

**Figure 8:**
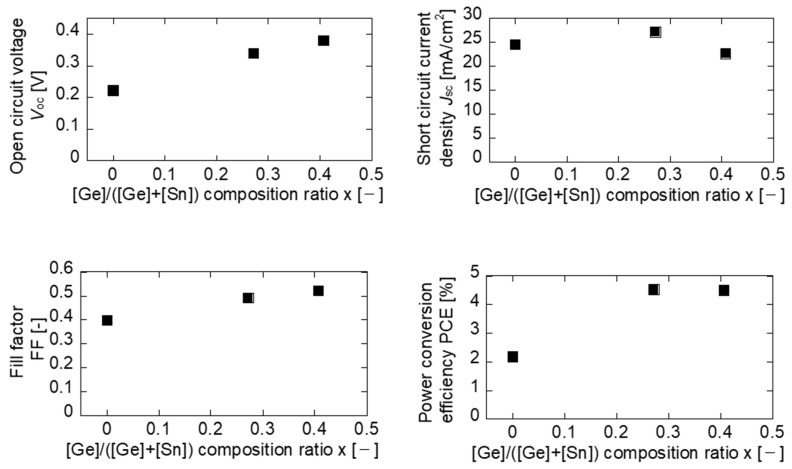
The x-dependence of photovoltaic properties in Na-doped CTGS solar cells.

Next, we analyze the dependence of the photovoltaic properties of the fabricated Na-doped CTGS solar cells on x. The x-dependences of *V*_oc_, *J*_sc_, fill factor (FF), and PCE of the Na-doped CTGS are shown in Figure 8. The values of *V*_oc_ at x = 0.27 and 0.41 are higher than that at x = 0.00, and the highest *V*_oc_ of 0.38 V is obtained at x = 0.41. This increase in *V*_oc_ is attributed to the widening of the band gap due to Ge alloying. In contrast, *J*_sc_ maximizes at x = 0.27 and decreases at x = 0.41. The decrease in *J*_sc_ is presumably due to the decrease in the current caused by the increased CTGS band gap. The FF exceeds that at x = 0.00 when both x = 0.27 and 0.41. The PCE of the CTGS solar cells with x = 0.27 and 0.41 is approximately 4.5%, representing a sufficient increase over the efficiency values obtained for the CTS with x = 0.00 by adding Ge and forming a solid solution CTGS.

## 4. Conclusions

In this study, CTGS thin films were fabricated with a controlled [Ge]/([Ge] + [Sn]) composition ratio x by combining the co-evaporation and sulfurization methods. During the process of fabricating CGS/CTS stacked precursors using the co-evaporation method, the x-value of CTGS was controlled by varying the deposition time of CGS. Furthermore, NaF deposition and sulfurization on the precursor increased the grain size of CTGS, which is similar to previously reported results for CTS. In the solar cell device constructed using the fabricated CTGS thin film, *J*_sc_ and *V*_oc_ improved significantly with Na doping owing to the crystal-growth-promoting effect of Na on CTGS.

## Figures and Tables

**Table 1:** Reported fabrication methods of CTS, CGS, and CTGS thin-film solar cells; range of [Ge]/([Ge] + [Sn]) composition ratios; and PCEs.

Precursor	Method	[Ge]/([Ge] + [Sn])	PCE [%]	Ref.
Soda lime glass (SLG)/Mo/Cu-SnS_2_/NaF	Sputtering and annealing with S and SnS	0	5.1	[11]
SLG/Mo/Cu/SnS_2_/NaF	Sputtering and annealing with S	0	5.2	[12]
SLG/Mo/Ge, Sn, and Cu laminated layers	Sputtering and annealing with S in a closed tube	0–1.0	~2	[13]
SLG/Mo/Ge/Cu–SnS_2_	Sputtering and annealing with S and SnS_2_	0–0.58	5.6	[14]
SLG/Mo/Cu–Sn	Co-sputtering and annealing with S and GeS_2_	0, 0.17	6.0	[15]
SLG/Mo/Cu–Sn	Co-sputtering and annealing with S and GeS_2_	Graded band gap structure	6.7	[16]
SLG/Mo/Sn/Ge/Cu	Sputtering and annealing with S	0.061–0.110	2.14	[17]
SLG/Mo/Cu(Sn,Ge)	Electrodeposition and annealing with S and GeS	0.83	0.7	[18]
SLG/Mo/Cu/Ge	Evaporation and annealing with S	1.0	1.70	[19]
SLG/Mo/Cu–Ge	Annealing of Cu-Ge alloy prepared by combustion method with S	1.0	2.67	[20]

**Table 2:** Composition ratios (x) of CGS/CTS stacked precursor and sulfurized CTGS measured using XRF. Sulfurized CTGS was designated as Na-doped CTGS and Na-free CTGS, respectively, depending on the presence or absence of NaF during sulfurization.

	*t*_CGS_ [h]	[Cu]/([Ge] + [Sn])	[Ge]/([Ge] + [Sn]) x	[S]/([Cu] + [Ge] + [Sn])
CGS/CTS precursor	0.0	1.58	0.00	1.07
	1.0	1.67	0.33	1.00
	1.5	1.68	0.45	0.98
Na-doped CTGS	0.0	1.60	0.00	1.15
	1.0	1.91	0.27	1.26
	1.5	1.94	0.41	1.16
Na-free CTGS	0.0	Peeled off	Peeled off	Peeled off
	1.0	1.80	0.32	1.07
	1.5	1.81	0.44	1.07

## Data Availability

Data are contained within the article.
